# Study of atherogenic lipid profile, high sensitive C-reactive protein neurological deficit and short-term outcome in stroke subtypes

**Published:** 2016-07-06

**Authors:** Aparna Pandey, Amit Shrivastava, Ashok Solanki

**Affiliations:** 1 Department of Biochemistry, Narsinhbhai Patel Dental College and Hospital, Visnagar, India; 2 Department of Biochemistry, Medanta-The Medicity, Gurgaon, ‎India‎; 3 Department of Physiology, AMC MET Medical College, L.G. Hospital, Maninagar, Ahmedabad, India

**Keywords:** Stroke, Lipid Profile, Ischemic Stroke

## Abstract

**Background:** Stroke is one of the most frequent causes of death and disability worldwide and has significant clinical and socioeconomic impact. Hyperlipidemia and inflammation play major roles in atherothrombosis and in stroke. This study is conducted to compare the high sensitive C-reactive protein (hs-CRP) levels and the lipid profile parameters between stroke patients and control group and demonstrate correlation between markers, neurological deficit, and short-term outcome.

**Methods:** We have studied a total 162 patients according to inclusion criteria. Serum level of hs-CRP and lipid profile estimated and correlated with neurological deficit and short-term outcome.

**Results:** We found stroke patients had significantly higher levels of hs-CRP, total cholesterol (TC), triglyceride (TG), low-density lipoprotein (LDL), and low level of high-density lipoprotein (HDL) than control. When we compared ischemic and hemorrhagic stroke (HS), data show increased level of triglyceride, LDL and HDL, and decreased the level of hs-CRP in ischemic stroke group than HS group. However, the National Institutes of Health Stroke Scale (NIHSS) score significantly higher in HS as compared to ischemic stroke at the time of admission and on the 7^th^ day.

**Conclusion:** Thus, continuous clinical observation is necessary for clear differentiation of those changes. Furthermore, the determination of some reliable soluble markers of neuronal damage in blood and cerebrospinal fluid in the early infarction period would be much easier and more useful for tracking the course and prognosis of the disease and for any appropriate therapeutic approach.

## Introduction

Stroke is one of the most frequent causes of death and disability worldwide and has significant clinical and socioeconomic impact. While hyperlipidemia and inflammation play major roles in atherothrombosis, there has been controversy regarding the relative contribution of these processes to stroke as compared to coronary heart disease.^1^ However, the relationship between atherosclerosis and elevated serum lipids is well established and aggressive treatment of dyslipidemia decreases the risk of stroke.^2^ Recent studies have shown that distribution of serum triglycerides (TGs) and total cholesterol (TC) within major lipoprotein classes majorly contribute to the development of atherosclerosis, which is the precursor to stroke. Hypercholesterolemia is a moderate risk factor for stroke. Elevated plasma concentration of low-density lipoproteins (LDL) and low high-density lipoprotein concentration (HDL) is associated with an increased risk of atherosclerosis.^3^

High sensitive C-reactive protein (hs-CRP) is a sensitive marker of inflammation and tissue injury in the arterial wall.^4^^,^^5^ Hs-CRP is a glycoprotein produced by the liver and plays a key role in the development of atherosclerotic disease in cerebral circulation.^6^^-^^9^ Hs-CRP is now the forerunner in the hunt for inflammatory markers and is subject to intensive research in numerous studies worldwide. Even though both hs-CRP and lipid profile parameters have a key role in initiation and progression of atherosclerosis; however, there is no data is available regarding the correlation of these two entities with respect to the risk stratification of stroke subtypes. Therefore, this study is conducted to compare the hs-CRP levels and lipid profile parameters between stroke patients and control group and demonstrate correlation between biomarkers with neurological deficit or short term outcome.

## Materials and Methods

A total of 200 patients with suspected stroke consecutively admitted to emergency department in which 162 patients with confirmed stroke were recruited in the stroke unit of Sri Aurobindo Institute of Medical Sciences, Indore, India. Subsequently, type of stroke identified by trained neurologist on the basis of clinical examination and a computed tomography (CT) scan of the brain and classified them as ischemic and hemorrhagic stroke (HS) subtypes. Criteria for exclusion were stroke more than 72 hours, peripartum stroke, HS or ischemic brain infarctions in the course of cerebral hemorrhage and concomitant major cardiac, renal, hepatic, and tumor diseases potentially interfering with standardized assessment of neurological or neuropsychological status. The inclusion criteria for subjects in the study group were adult stroke (age > 21 years) and within 72 hours of admission. This study was approved by the ethics committee of hospital, and all patients or relatives gave written informed consent.

***Biochemical examinations***

Serial venous blood samples were collected at admission, at 7^th^ day. To determine serum hs-CRP and lipids levels, blood samples were taken following a 10-12 hour fast. Blood was allowed to clot at room temperature, and serum was obtained immediately by centrifugation at 3500 revolutions per minute (rpm) for 10 minutes. Serum was aliquoted into plastic tubes and stored at -27 °C until assayed. Serum hs-CRP was assayed by turbidimetric test using Roche reagent kit. TC and serum TG were estimated by commercial available standard kits of Roche Diagnostics. HDL-cholesterol (HDL-C) was measured by a reagent kit of Sigma Diagnostics and LDL-cholesterol (LDL-C) by a reagent kit of Accurex diagnostics. All parameters were assayed in Hitachi 917 auto-analyzer, according to the instructions of the manufacturer.

***Neurological assessments***

All subjects underwent a standardized neurological examination at the time of admission and at 7^th^ day in the stroke unit. The neurological deficit was quantified by the use of the National Institutes of Health Stroke Scale (NIHSS). We performed comprehensive neuropsychological examinations in all patients with a first-ever stroke event and without any clouding of consciousness or severe disorders of attention.

Demographic, clinical and laboratory frequency variables were calculated. Statistical analyses were performed with SPSS for windows (version 17, SPSS Inc., Chicago, IL, USA). Parameters value expressed as a mean ± standard deviation (SD). Statistical significance of the difference between the categorical variables was tested with the chi-square test. Statistical significance for the intergroup difference was assessed by Student’s t-test. To study the correlation between quantitative variables, Pearson test was used. A probability value P < 0.050 was considered statistically significant. In all cases, a 95% confidence level was used.

## Results

The demographic and clinical patient data with cerebral infarction are shown in table 1. 

**Table 1:** Demographic profile

**Parameters**	**Stroke subjects (n = 162)**	**Control (n = 101)**
Sex (Females/Males)	65/97	34/67
Age (year) (mean ± SD)	58.96 ± 12.37	55.54 ± 7.67
Hypertension (mmHg)	118/44	30/61
Smokers (Yes/No)	54/108	26/75
AF (Yes/No)	42/130	21/80
DM (Yes/No)	58/104	27/74
Alcohol (Yes/No)	60/102	26/75

SD: Standard deviation; AF: Atrial fibrillation; DM: Diabetes mellitus

There were no differences in terms of age or sex as shown in table 1. 118 (72%) patients had hypertension, 54 (34%) were smokers, 42 (25%) had atrial fibrillation, 58 (36%) had diabetes mellitus (DM), and 60 (37%) were alcoholic. The study comprised 162 patients that include 100 patients of ischemic stroke and 62 of HS. 101 healthy subjects in the age of 30-58 years were also investigated on similar line and served as control groups. The mean age of the stroke patients were 58.96 ± 12.37 and controls were 55.54 ± 7.67.

Table 2 shows biomarkers and lipid profile status of subject (stroke patients) and control. Stroke patients show significantly elevated level of hs-CRP (4.11 ± 2.00 vs. 0.88 ± 4.90), TC (263.58 ± 38.15 vs. 251.95 ± 35.67), TG 208.13 ± 35.30 vs. 116.65 ± 34.40), LDL-C (191.40 ± 36.40 vs. 88.4 ± 13.6) and significantly decreased level of HDL-C (29.33 ± 5.60 vs. 46.90 ± 12.11) than control. 

Table 3 indicates comparison of biomarker and lipid profile status between ischemic and HS group. Data show increased level of TG (229.00 ± 23.07 vs. 99.34 ± 37.17), LDL-C (31.94 ± 5.17 vs. 25.33 ± 3.54) and HDL-C (31.94 ± 5.17 vs. 25.33 ± 3.54) and decreased the level of hs-CRP (2.90 ± 1.70 vs. 5.60 ± 1.10) in ischemic group then hemorrhagic group. However, NIHSS score significantly higher in hemorrhagic group as compared to ischemic group at the time of admission (13.48 ± 7.10 vs. 21.63 ± 1.74) and after 7^th^ day (12.30 ± 7.81 vs. 25.58 ± 3.30), respectively.

**Figure 1:**
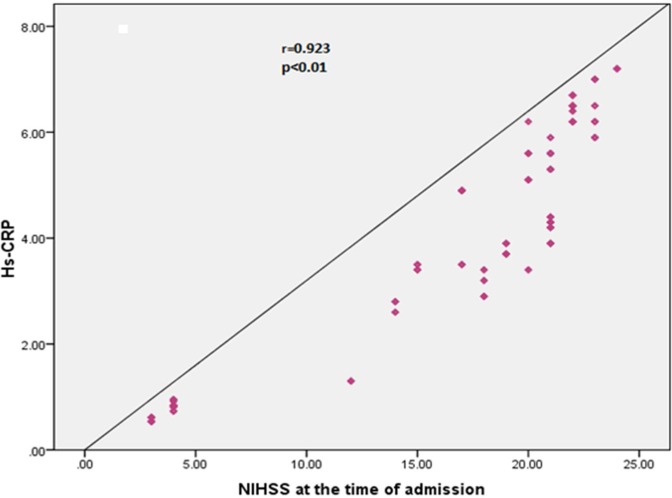
Correlation between high sensitive CRP and neurological deficit

**Figure 2:**
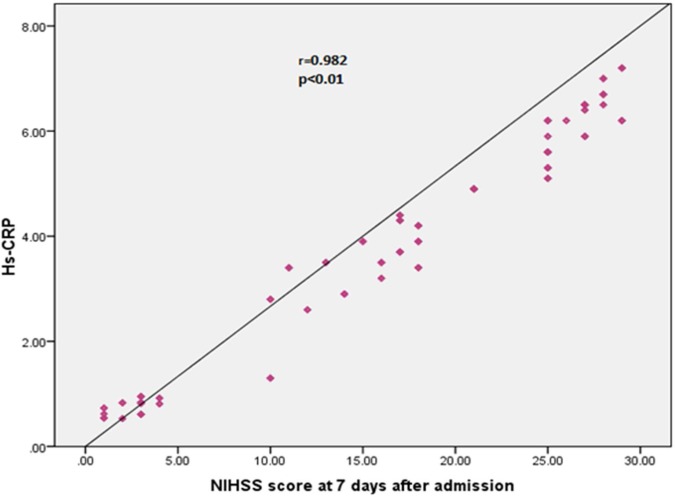
Correlation between high sensitive CRP and neurological deficit

We found highly significant positive correlation between hs-CRP and NIHSS score [at the time of admission (Figure 1) (r = 0.923, P < 0.010) and 7^th^ day of admission (Figure 2) (r = 0.982, P < 0.010)]. No statistically significant correlations were found between lipid profile and NIHSS score (at the time of admission and 7^th^ day of admission).

hs-CRP: High sensitive C-reactive protein; NIHSS: National Institutes of Health Stroke Scale

**Table 2:** Comparison of biomarkers and lipid status between stroke patients and control

**Parameter**	**Subject (n = 162)**	**Control (n = 101)**	**t value**	**P**
hs-CRP (mg/L) (mean ± SD)	4.11 ± 2.00	0.88 ± 4.90	7.106	< 0.001***
TC (mg/dL) (mean ± SD)	263.58 ± 38.15	251.95 ± 35.67	14.84	< 0.016**
TG (mg/dL) (mean ± SD)	208.13 ± 35.30	116.65 ± 34.40	20.300	< 0.001***
LDL-C (mg/dL) (mean ± SD)	191.40 ± 36.40	88.4 ± 13.6	27.146	< 0.040*
HDL-C (mg/dL) (mean ± SD)	29.33 ± 5.60	46.90 ± 12.11	15.501	< 0.010**

HDL-C: High density lipoprotein-cholesterol; LDL-C: Low density lipoprotein-cholesterol; hs-CRP: High sensitive C-reactive protein; TC: Total cholesterol; TG: Triglycerides; SD: Standard deviation

* Significant,

** Highly significant,

*** Extremely significant, Degree of freedom (df) = 262

**Table 3:** Comparison of C-reactive protein‎ (CRP) and lipid profile between ischemic with hemorrhagic stroke (HS) patients

**Parameter**	**Ischemic stroke (n= 100)**	**HS (n= 62)**	**t value**	**P**
hs-CRP (mg/L) (mean ± SD)	2.90 ± 1.70	5.60 ± 1.10	14.73	0.001***
TG (mg/dL) (mean ± SD)	229.00 ± 23.07	99.34 ± 37.17	13.55	0.001***
LDL-C (mg/dL) (mean ± SD)	222.17 ± 21.80	84.11 ± 8.63	8.95	0.005**
HDL-C (mg/dL) (mean ± SD)	31.94 ± 5.17	25.33 ± 3.54	17.37	0.001***
TC (mg/dL) (mean ± SD)	296.70 ± 20.54	241.51 ± 30.43	11.83	0.004**
NIHSS score at the time of admission	13.48 ± 7.10	21.63 ± 1.74	13.93	0.050*
NIHSS score at 7^th^ day of admission	12.30 ± 7.81	25.58 ± 3.30	11.83	0.050*

HDL-C: High density lipoprotein-cholesterol; LDL-C: Low density lipoprotein-cholesterol; hs-CRP: High sensitive C-reactive protein; TC: Total cholesterol; TG: Triglycerides; NIHSS: National Institutes of Health Stroke Scale; HS: Hemorrhagic stroke; SD: Standard deviation

* Significant,

** Highly significant,

*** Extremely significant, df = 161

hs-CRP: High sensitive C-reactive protein; NIHSS: National Institutes of Health Stroke Scale

## Discussion

Various studies showed that hyperlipidemia and inflammation may accelerate process atherothrombosis and worsening of stroke outcome. In this study, a mean value of inflammatory marker hs-CRP in total stroke patients was higher as compared to control groups. Winbeck et al.,^10^ Di Napoli et al.,^11^^,^^12^ and Eikelboom et al.^13^ also reported that the level of hs-CRP, a peripheral marker of inflammation, has consistently been observed to be related to the risk of cerebrovascular and cardiovascular events, and it is systematically elevated in the circulation of patients after acute stroke. Rost et al.^14^ recently have shown that elevated hs-CRP levels independently predict the risk of future stroke and transient ischemic attack (TIA) in the elderly. Wakugawa et al.^9^ found that hs-CRP may be involved in each of these stages by direct influencing processes such as complement activation, apoptosis, vascular cell activation, lipid accumulation, and thrombosis.

Atherosclerosis, one of the major causes of cerebrovascular disease is considered to involve the inflammatory system. HS-CRP, widely known to be an inflammatory marker, was detected in atherosclerotic plaques. It may be explained by the fact that, inflammation plays a central role in all phases of arthrosclerosis, from the initial recruitment of circulating leukocytes to the arterial wall to the rupture of unstable plaques, which results in the clinical manifestations of the disease. When hs-CRP levels were compared in different subtypes of stroke, the mean hs-CRP level was more in HS than ischemic stroke. Mishra et al.^15^ also found significantly increased levels of hs-CRP in HS patients although these results are different from those of Wakugawa et al.^9^ in the Hisayama study in which they observed no clear association between hs-CRP levels and HS occurrence. Ridker et al.^16^ considered hs-CRP as marker of low-grade vascular inflammation, which is a key factor in the development and rupture of atheromatous plaque. Present data suggested that inflammation plays a key role in the development of brain injury after hemorrhage. HS-CRP is normally absent in the blood. The presence of acute inflammation with tissue destruction within the body stimulates its production, allowing the inflammation to be confirmed.

In present study, total stroke patients showed highly significant rise in TC as compared to control. Stroke is usually caused by a sudden blockage to the arteries carrying blood to parts of the brain. When there is an excess of cholesterol in the artery walls, narrowing of arteries or even a complete blockage can occur in the artery. At narrow points in the arteries, blood clots can form and either block the arteries or break off. Dayton et al.^17^ did not find clear correlation between serum cholesterol levels and the risk of stroke. Gorelick and Mazzone^18^ showed a U-shaped relation between the level of serum TC and the risk of stroke of all types, derived from an inverse association with HS and a direct association with ischemic stroke. Konishi et al.^19^ found that the possible differences in the effects of cholesterol at different vascular sites could lead to the complex association between serum cholesterol levels and stroke. The origin of the internal carotid artery is probably the most common site of atherosclerosis that leads to TIA or stroke. A highly significant decrease in TC was observed in stroke patients with hemorrhage as compared to ischemic stroke patients. 

Iso et al.^20^ found an inverse relation between cholesterol level and HS but a positive association with non- HS. Tanizaki et al.^21^ in their study found that lower TC was an additional risk factor for cardioembolic infarction in women. Iribarren et al.^22^ confirmed a positive association between low serum cholesterol level and intracerebral hemorrhage in elderly men. The mechanisms explaining how low TC could promote HS still remain under investigation. In stroke prone rats, low cholesterol level and abnormal erythrocytes fragility have been reported. It has been hypothesized that such a phenomenon in the endothelial cell could lead to arterionecrosis. Moreover, very low cholesterol could promote cerebrovascular endothelium fragility, which could generate angionecrosis and intracerebral hemorrhage in a context of high pressure.

In total stroke serum, TGs were higher than control in the present study indicating a directly proportional relationship between TH levels and risk of stroke. Hachinski et al.^23^ found a positive correlation of serum TG levels with patients suffering from atherothrombotic stroke and TIAs as compared to control subjects. Elevated TGs are markers of elevated levels of lipoprotein remnants [very LDL [VLDL]) which are thought to contribute to plaque build-up. The present study data showed that significant low level of TGs in HS subtypes. Two population-based studies found a positive relationship between low TG and the raised incidents of HS appeared primarily in men and in older age groups, respectively.^22^^,^^24^ On the basis of data present study hypothesized that the association between low TG level and HS may appear among in individuals with an elevated cerebrovascular risk profile and have particular sensibility to arterial injury. 

This study revealed a positive association between LDL-C and the risk of ischemic stroke. Pedro-Botet et al.^25^ and Hachinski et al.^23^ have found positive correlation between LDL-C levels and risk of ischemic stroke. Treatment with cholesterol-lowering medications and changes in LDL-C level over time may have attenuated the risk in stroke population, and lipid measurements at several points may be a better marker of stroke risk.^26^ Kar et al.^27^ proved that there was an impressive correlation of increased LDL-C to the atherosclerosis. Varying levels of serum LDL-C are responsible for deferring vascular pathogenesis of underlying stroke. LDL-C is believed to be the most atherogenic lipoprotein. 50% of cholesterol in plasma is found in the form of LDL-C. LDL delivers cholesterol to tissues via a specific high affinity LDL receptor, which controls the uptake of cholesterol by cells. An excess amount of LDL-C in the blood builds up on the inside of the blood vessel walls, making it more difficult for blood to flow freely. This increases the risk of developing stroke. When LDL-C levels were compared in different subtypes of stroke, the mean LDL-C levels were significantly low in HS than ischemic stroke in the present study. Noda et al.^28^ report an association between low levels of LDL-C with an increase in the risk of fatal intra-parenchymal intracerebral hemorrhage in a Japanese population-based cohort. Goldstein^29^ found that the general population, having low, usual TC and LDL-C appear to be associated with a higher risk of brain hemorrhage. The data of present study are in agreement with the studies of Noda et al.^28^ and Goldstein^29^ that the low LDL-C appears to be associated with higher risk of brain hemorrhage. 

In the present study, it was found that the stroke patients had low level of HDL-C than that of the control group. A study by Kar et al.^27^ proved a strong positive correlation of increased LDL-C and decreased HDL-C to the atherosclerosis. Wannamethee et al.^30^ reported that higher levels of HDL-C were associated with a significant decrease in the risk of non-fatal stroke. Bloomfield Rubins et al.^31^ revealed that gemfibrozil, a fibrate that raises HDL-C levels, reduced the risk of ischemic stroke by 31% in men with congenital heart disease, supporting the idea that HDL-C levels may be important in the pathogenesis of ischemic stroke. Albucher et al.^32^ studied 90 young ischemic coronary disease patients and concluded that HDL-C was the only serum lipid index to be associated to an increased risk of stroke. Low HDL-C must be considered in the care and management of young patients regardless of the detectable presence of atherosclerosis. HDL-C is important for removal of cholesterol from the peripheral tissues to the liver, metabolism of chylomicrons and VLDL-cholesterol. HDL-C affects the metabolism and transport of other lipid fractions. A non-significant alteration in HDL levels was reported in comparison of HS and ischemic stroke patients. 

When disability score was compared in different subtypes of stroke, the score was more in HS than ischemic stroke. Andersen et al.^33^ and Jorgensen et al.^34^ found that strokes are generally more severe in patients with HS and the ratio between HS and ischemic stroke closely related to stroke severity. Corbin et al.^35^ figures out those patients with HS are generally considered to be at high risk for mortality compared to patients with infarcts. Franke et al.^36^ linked the excess mortality to the generally more severe strokes in patients with HS, whereas stroke type per se was considered to have no influence on mortality-The extent of the injury and initial stroke severity was regarded decisive. Chiu et al.^37^ found that intracerebral hemorrhage is an independent predictor of poor neurologic outcome, nearly doubling the odds of long-term disability. However, intracerebral hemorrhage is not associated with higher mortality compared with ischemic stroke after adjusting for initial stroke severity and other baseline characteristics. The present ﬁndings supports and extend previous studies on functional outcome after ischemic and HS and concluded that patients with HS patients had a more functional impairment (at the time of admission and 7^th^ day of admission).

## Conclusion

In the last decade, multiple techniques were explored and were found useful in the evaluation of the neurological deficit as well as in recovery prediction after the application of therapy in patients with brain ischemia. 

Modern neuroradiological techniques, such as CT and nuclear magnetic resonance, are useful in identification of locality and extent of the ischemic lesion, but in the relatively late period, when lesions are mostly formed. Furthermore, in the early period after the onset of a cerebral insult, it is difficult to clinically distinguish and evaluate reversible from irreversible changes in the brain. Thus, continuous clinical observation is necessary for clear differentiation of those changes. Moreover, the determination of some reliable soluble markers of neuronal damage in blood and cerebrospinal fluid in the early infarction period would be much easier and more useful for tracking the course and prognosis of the disease and for any appropriate therapeutic approach.

## Notes:

**How to cite this article:** Pandey A, Shrivastava A, Solanki A. Study of atherogenic lipid profile, high sensitive C-reactive protein neurological deficit and short-term outcome in stroke subtypes. Iran J Neurol 2016; 15(3): 146-52.
